# “Kuteteza”: A community-engaged COVID-19 Prevention and Protection Initiative in Southern Malawi

**DOI:** 10.12688/wellcomeopenres.20789.1

**Published:** 2024-02-12

**Authors:** Donnie Mategula, Ana Ibarz-Pavón, Melody Sakala, Marlen Chawani, Henry Sambakunsi, Mphatso D. Phiri, Latif Ndeketa, Mwiza Sambo, Wisdom Shonga, Clara Sambani, Titus Divala, Steve Vinkhumbo, Dominic Nkhoma, Robert Mataya, Wongani Nyangulugu, Sepeedeh Saleh, Kuteteza study group

**Affiliations:** 1 Malawi-Liverpool-Wellcome Trust Clinical Research Programme, Blantyre, Southern Region, Malawi; 2 Liverpool School of Tropical Medicine, Liverpool, UK; 3 Kamuzu University of Health Sciences, Blantyre, Malawi; 4 University of Liverpool, Liverpool, England, UK; 5 Society of Medical Doctors, Lilongwe, Malawi; 6 Malawi Ministry of Health, Lilongwe, Malawi

**Keywords:** Malawi, COVID-19, Community, Engagement, Public Health

## Abstract

**Background:**

The COVID-19 epidemic in Malawi involved almost 90,000 recorded cases and 2,638 deaths. Data suggest that the Malawi epidemic developed largely in urban settings, but the extent of rural spread – exposing older adults in particular – is unclear. This concern led us, to develop the ‘Kuteteza’ partnership project: a community-based Public Health intervention involving the shielding of older adults and additional strategies to mitigate COVID-19 impacts in rural Malawi. We now present the results of a realist project evaluation.

**Methods:**

Clinicians, Public Health professionals, and researchers collaborated with government and district level staff in two Malawi districts. After engagement with local structures, willing older adults (>60 years) and their communities made arrangements for supported 'shielding'. Masks, handwashing stations, and soap were also provided, and government partnerships allowed additional support for vulnerable groups. Finally, context-responsive community engagement reinforced COVID-19 prevention during peak risk periods. Our realist evaluation included collation of anonymised descriptive data and qualitative structured observations and focus groups – involving community members and volunteers – in each setting.

**Results:**

The project involved 25 villages. Multi-level stakeholder engagement was pivotal in developing and establishing the work, and strong District Health Office support was integral to implementation. Team members contributed to national meetings, aligning the project with national guidance and stakeholder actions. Established partnerships with relevant ministries resulted in the incorporation of shielding in the Malawi COVID-19 response plan. In Kuteteza villages, handwashing stations and soap were used, and there was awareness of COVID-19 prevention measures. Further experiences and evaluation results are presented, including learning points for future outbreak responses.

**Conclusions:**

Through effective stakeholder engagement and contribution to national response strategy, the Kuteteza project raised awareness and supported populations at a critical time in the pandemic. These approaches can be incorporated in future epidemic and emergency responses.

## Plain Language Summary

During the COVID pandemic in Malawi, cases were seen first and most often in the cities. Serious concerns were raised about how the pandemic might affect more rural populations, with poorer access to healthcare, greater income insecurity, and higher numbers of older adults (aged over 60 years). In an effort to prevent the worst of the expected impacts for older adults living in the villages, we formed a partnership of health workers, researchers, and policymakers, setting up the 'Kuteteza' response project (meaning ‘to protect’ in the Chichewa language).

The project worked with community members in 25 villages, supporting communities to enable 'shielding' of older adults: minimising their social mixing whilst also providing items such as handwashing stations, masks, food parcels and regular supplies of soap. Volunteers and health workers engaged with residents, spreading evidence-based information about COVID and feeding back community insights. This paper describes the results of a project evaluation – reporting numbers of villages and people involved, observations from participating villages, and insights from community members in focus groups.

One of the mains strengths of the project was in how the team worked together and with communities themselves, making sure actions were in line with national and regional policy and guidance and that it could quickly adapt to evolving needs as the pandemic progressed. We recommend these approaches for future responsive health projects, and present the outcomes of our evaluation for learning and discussion.

## Introduction

Malawi saw its first cases of severe acute respiratory syndrome coronavirus 2 (SARS-CoV-2) in April 2020 ^1^: these constituted a small wave in July–August 2020, which was then followed by larger waves in January–February 2021, July–August 2021, and December 2021–January 2022 ^2^. Case numbers were relatively high with over a thousand cases per day at the height of the second and fourth waves, and over 85 000 confirmed cases nationally by February 2022 ^2^. Epidemiological patterns of the disease were like those seen in the rest of the world, with case severity and mortality rates in increasing with age, and higher in males than females ^3, 4^.

The main disease epicentres in Malawi were the urban cities of Blantyre and Lilongwe, which experienced the highest numbers of confirmed cases, hospitalisations, and deaths from COVID-19, particularly in the first three waves. Of the 61 846 confirmed cases by November 2021, approximately 50% were in the cities of Blantyre or Lilongwe, with 46% of the 2302 confirmed deaths in Malawi from COVID-19 also occurring in these two cities ^5^.

Following initial importations to Malawi and the initial spread through largely urban events, which drove the first wave, there was relatively more transmission to rural areas across the country in the third and fourth waves ^3, 6^. Concerns here lay in the often-severe barriers to healthcare access in these regions as well as in the much greater presence of older adults in these areas, with over 90% of Malawi’s older adult population residing in rural communities ^7– 10^.

In Malawi, the government implemented a coordinated multisectoral approach to COVID-19 response through establishment of operational clusters that comprised of multidisciplinary experts and development partners ^11^. The protection cluster was specifically mandated to prevent and address the impacts of the COVID-19 outbreak through coordination and support to advocate for inclusion of specific rights, needs and vulnerabilities of susceptible groups including older adults ^12^. This cluster was led and coordinated at the central level by the Ministry of Gender and UNICEF, and its activities were implemented in collaboration with various social protection partners at national, district and community levels. The cluster activities included providing policy guidance, risk communication and community engagement, and social protection support ( *e.g.,* cash transfers, food items, and protective equipment) ^13^. Whilst the overall objectives of the cluster included addressing the needs of older adults as one of the vulnerable groups, no specific activities targeting older adults were indicated or budgeted for in the first national COVID-19 response plan.

Public Health approaches to preventing the spread of COVID-19 must be multifaceted and context-aligned for maximum effectiveness. One proposed element of such a public health approach is shielding: limiting the contact between those at high risk of developing severe disease (such as older adults) and the general population to reduce the number of severe cases in the population, thereby also protecting health systems from undue pressure ^14– 16^. This could be arranged in various ways, such as at individual household or neighbourhood level, with the key elements of the approach including provision of adequate facilities and maintenance of minimum living standards for shielded individuals and the separate isolation of any potentially affected individuals away from others as soon as possible ^17, 18^.

There has been some evidence on the shielding of certain vulnerable groups, particularly early in national COVID-19 epidemics ^18– 20^, but no global consensus on its role in the pandemic response. Controversies surrounding the use of shielding in high-income countries have occurred due to concerns around the exclusion of the most vulnerable, and serious consequences of increased spread in settings where large numbers of high-risk individuals are brought together ^21^. It is therefore evident that any adoption of shielding approaches must be carefully considered, locally contextualised, and must only be implemented in a fully participatory way with empowered community engagement throughout ^22^.

Expert guidance on the subject from the Social Science in Humanitarian Action Platform (SSHAP) partnership ^18^ emphasises the need for situational adaptation to allow co-development of any such interventions, together with community members; the importance of informed choice, with the option to shield being open to individuals and their families and households; and the need for cross-sectoral management throughout. They recommend that shielding should be part of a wider response that ensures basic water, sanitation and hygiene (WASH) facilities, COVID-19 awareness and services, safeguarding, and data collection to allow the assessment of any benefits and potential risks ^18^.

‘Kuteteza’ (meaning ‘to protect’ in vernacular Chichewa) was a project using community engagement and participation in villages across two districts of Southern Malawi, to combine shielding approaches with wider COVID-awareness, and provision of WASH and basic facilities for those in most need. In building a coordinated response involving researchers, environmental, public health, and government teams, and community members on the ground, we developed an engaged, multi-component approach to protecting those potentially most at risk in the population from the direct and indirect harms of COVID-19 in Malawi.

## Methods

### Setting and study population

The project engaged with stakeholders at three tiers: national, district, and community levels. Nationally, we collaborated with the Department of Disability and Elderly Affairs and engaged with the Ministry of Health, through various national committees involved in the COVID-19 response. At the district level we worked particularly in Blantyre and Mangochi districts, both in the Southern region of Malawi, engaging with existing District Health Organisations, contributing to regular fora and responding to expressed needs at this level. In view of the responsive nature of the project to the emerging pandemic, districts selected for inclusion were on the basis of existing research and organisational networks, to allow effective engagement with health systems and rapid project rollout.

Blantyre, the economic capital of Malawi, has a population of about 800,264 and a relatively high population density compared with other cities in the county. Mangochi district, also in southern Malawi, has a mostly rural economy driven by agriculture and tourism, with an adult population of around 516, 976 ^10^. At the community level, we worked in twenty villages across Blantyre, located in rural areas outside the city limits. Mangochi district, also in southern Malawi has a mostly rural economy driven by agriculture and tourism, with a population of around 610,239. Here, the intervention focused on five villages surrounding the district’s town centre. In both districts, community members worked with project volunteers to identify residents over the age of 60 who – with their consent – were then included for (voluntary) participation in the study.

The focus group discussion (FGD) guide, additional data collection tools, participant information sheets and consent forms can be found as *Extended data* ^23^.

### COVID response project

The project involved three phases, described below.

Phase 1 - A cross-sectional mapping exercise of COVID-19 stakeholders at national and district levels. This helped us to understand the need for a COVID-19 response in rural areas, with particular attention to protecting those with a high risk of severe outcomes.

Phase 2 - Implementation of the Kuteteza package. The intervention involved mitigating population-level morbidity and mortality from COVID-19 disease in rural areas of Malawi through a programme of supported ‘shielding’ with the assistance of community health workers, known in Malawi as Health Surveillance Assistants (HSAs), and village volunteers.

Local activities incorporated:

- Preparatory work, training local volunteers in community engagement approaches and COVID information and building communication links in each district between researchers, HSAs and volunteers.
- Awareness raising involving spreading of basic, evidence-based COVID information through posters and leaflets in public spaces.
- Enumeration of (voluntarily self-identified) older people in each village – defined as those aged over 60 years.
- Supported rearrangement of living spaces for these individuals and provision of basic necessities (food, water, etc.), to allow ‘shielding’ within a household area, minimising contact with the rest of the community. The two above steps were implemented across the whole study area following a local pilot in one village, with small adaptations as necessary prior to rollout.
- Provision of handwashing stations (Veronica buckets) to all 'shielding' households and regular provision of soap for use at these stations.
- Provision of face masks to public, for use in public transportation, health clinics, etc.
- Liaison with government departments and national committees to target available emergency resources (food packages) to most vulnerable residents in participating villages.

Phase 3 – Evaluation of the interventions using both qualitative and quantitative approaches. Results of this evaluation are described in the current paper.

### Evaluation study design

We utilised a case study approach to qualitative inquiry to help us understand experiences of the shielding initiative, identify any challenges, strengths and potential areas for improvement. A prospective ecological evaluation of the implementation was used to collect information on the process and outcomes, including understandings around COVID-19 and community and individual-level actions aimed at COVID-19 risk reduction. Details of evaluation methods are provided below.

***Data collection***

**Quantitative data**

Team members compiled an anonymised listing of village residents aged over 60 years who participated in shielding. For each participant we collected information on proposed shielding arrangements and on arrangements for the provision of basic necessities and alternative care for dependents, such as infants, where necessary.

Aggregated data on the total numbers of suspected and confirmed cases of COVID-19 among individuals entering the project were collected through HSAs. We also collated data on the number of community engagement activities conducted and quantities of items distributed to measure uptake of the strategy and other approved interventions ( *e.g.,* use of masks) in the community.

**Qualitative data**

Assessment methods included structured observations in each district and village, and district level focus group discussions (FGDs), involving residents, community volunteers and health surveillance assistants. Participant recruitment began on 20 ^th^ September 2021 and data collection started later in the same week, on 24 ^th^ September.

Table 1 summarises the qualitative tools and areas of evaluation.

**Table 1.** Summary of qualitative data collection tools and their aimed areas of evaluation. FGD, focus group discussion; HSA, Health Surveillance Assistant.

Activity	Participants	Participants
Structured observations	Assessments carried out on walks around village, incorporating key sites in the village and events – e.g. schools, places of worship	COVID-19 risk reduction actions underway in village environment: - Presence of Ministry of Health approved information and awareness materials - Availability of handwashing stations around the village - Mask wearing amongst residents and others. Following of additional COVID-19 prevention guidance
Village level focus groups (Two in each district – four in total)	Village residents – including older and younger members of the community (12 participants per FGD)	Communities’ perceptions around COVID-19 risks and responses taken, considering influence of the Kuteteza project in shaping these Assessment of intervention approaches and implementation including strengths, challenges, and opportunities for improvement
District level focus groups (One in each of the two districts)	District HSA teams, health volunteers, and village chiefs (9–12 participants per FGD)	Assessment of intervention, approaches, and implementation, including strengths, challenges, and opportunities for improvement

### Ethical considerations

The original project was a piece of responsive Public Health work. Shielding was implemented as part of the Public Health response to COVID-19, in close collaboration with the Ministry of Health and local health authorities. Participation in shielding elements was voluntary, and no information was required from those who opted into shielding, other than access to anonymised population level data. In terms of the qualitative evaluation, focus group participants were required to provide written informed consent, which was collected by trained field workers, and all data and documents were kept under the custody of the principal investigator in a locked cabinet, located in a restricted-access area. A named safeguarding lead was in place throughout the project, with various avenues of contact for participants to report any concerns, and structures for appropriate referral of any such reports. The project was sponsored and approved by the local ethics committee (College of Medicine Research Ethics Committee; COMREC) in Blantyre, Malawi (P.01/21/3246) on 7 ^th^ July 2021.

### Data analysis

***Quantitative data***

Data were collected through dedicated paper-based case report forms designed by the research team and entered onto an electronic database by dedicated data clerks. Descriptive analysis was conducted using R ^24^. Categorical variables are expressed as totals and percentages, while continuous variables are expressed as means, and defined by 95% confidence interval and standard deviation.

***Qualitative data***

Focus groups were conducted in Chichewa and facilitated by Malawian research team members. All focus groups were audio recorded (with consent from participants) and subsequently transcribed and translated into English by research assistants prior to uploading onto NVIVO for analysis by three members of the research team ^25^ [ Taguette is a free, open-source alternative to NVIVO]. Observation notes were also translated and entered onto NVIVO for analysis. Reflexive thematic analysis was used for analysing all the qualitative data as it is a well-described method and relatively easy for multiple researchers to work on together (with discussions of themes and codes as they developed) ^26^. Its theoretical flexibility also made it suitable for the current evaluation, with multiple sub-questions under investigation and different data sets from the two data collection methods used. Researchers created initial codes, then brought these to a group discussion for development and refinement and co-development of key themes with reference to the original data.

All qualitative and quantitative data were anonymised to preserve participants’ confidentiality and held on secure systems with access limited to only the necessary subset of researchers.

### Reflexivity statement

*The reflexivity statement* can be found as *Extended data* ^23^.

## Results

### Quantitative findings

A total of 1,640 individuals across 20 villages in Blantyre and 5 villages in Mangochi identified themselves as being over the age of 60 ( Table 2). This included 1,138 individuals in Blantyre (69.4%) and 502 in Mangochi (30.6%). Of these, 1,087 – 746 in Blantyre (68.6%) and 341 in Mangochi (31.2%) – agreed to participate in shielding ( Table 3).

**Table 2.** Summary of participants in Blantyre and Mangochi.

	BLANTYRE	MANGOCHI
**Total villages**	20	5
**Total Shielded**	746	341
**Total individuals with data**	478	267
**Mean age**	75 (95% CI: 73.2-77.4, sd 8.8)	72.3 (95% CI 71.2-73.4; sd 9.0)

**Table 3.** Summary of provision arrangements across Blantyre and Mangochi.

	WATER (n (%)) Blantyre Mangochi		FOOD (n(%)) Blantyre Mangochi		MEDICINES * (n(%)) Blantyre Mangochi		FIRE/FUEL (n(%)) Blantyre Mangochi	
**Adult family member**	157 (32.8)	184 (68.9)	158 (33.1)	190 (71.2)	39 (39.4)	65 (94.2)	153 (32.0)	185 (69.3)
**Adult non-family member**	0	1 (0.3)	0	1 (0.03)	3 (3.0)	0	0	1 (0.3)
**Child family member**	261 (54.6)	16 (6.0)	250 (52.3)	19 (7.1)	41 (41.4)	3 (4.3)	256 (53.6)	16 (6.0)
**Child non-family member**	0	1 (0.3)	1 (0.2)	1 (0.3	0	0	0	0
**Unknown**	60 (12.6)	65 (24.3)	69 (14.2)	56 (21.0)	16 (16.1)	1 (1.4)	69 (14.4)	65 (24.3)

* 99/478 in Blantyre and 69/267 in Mangochi known to need medicines

Individual-level data were available for 478 individuals (64.1%) from Blantyre and 267 (78.3%) from Mangochi. Of these, the mean participant age was 75 years in Blantyre and 72.3 in Mangochi. A total of 99 participants (20.7%) from Blantyre and 69 (25.8%) from Mangochi needed medication to treat chronic conditions ( Table 3), and 471 (98.5%) and 239 (89.5%) participants in Blantyre and Mangochi respectively, had dependents under their care ( Table 4).

**Table 4.** Summary of care arrangements for dependents of shielded individuals across Blantyre and Mangochi.

	DEPENDENTS (n (%))	
	Blantyre	Mangochi
**Remain with shielded person**	355 (98.6)	238 (94.8)
**Made other arrangements**	5 (1.4)	12 (4.7)

* 360/478 in Blantyre and 251/267 in Mangochi had dependents

**Figure 1.**
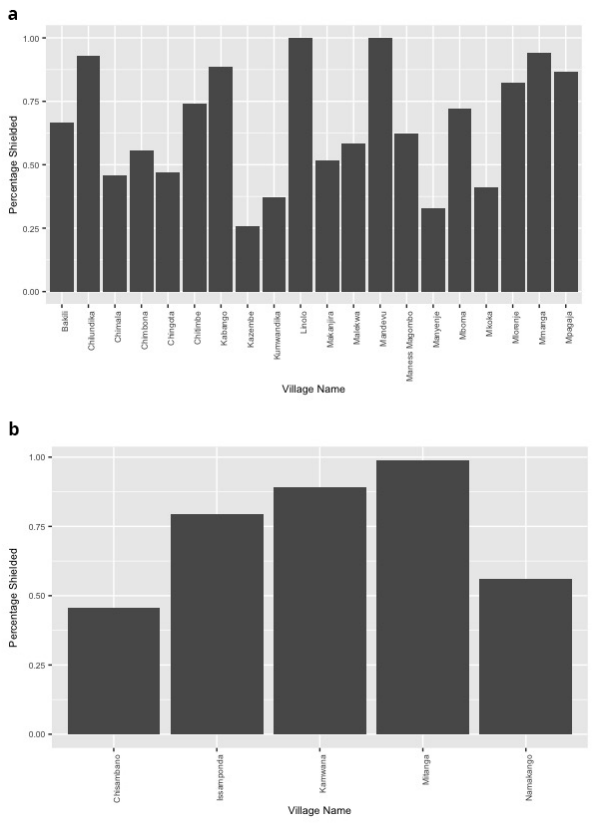
Percentage of eligible individuals that participated in shielding during the COVID-19 pandemic across villages in Blantyre ( **a**) and Mangochi ( **b**).

The uptake of shielding varied by village, ranging between 32.8% and 100% across villages in Blantyre and between 45.7% and 98.8% in Mangochi villages ( Figure 1). Across both settings, supplies of food, water, and fuels such as charcoal or firewood for the cooking fire were mostly arranged within the family. Still, there were important differences between the districts in this area. While in Mangochi, water, food, and fuels were supplied by adult family members in approximately 70% of cases, and medicines in 94.2% of those who required them, in Blantyre most supplies were arranged by children in the family. In more than 50% of participant households in Blantyre, children had responsibility for supplying basic necessities to the shielding individuals, including medicines.

Throughout the duration of the pilot, 120 individuals in the shielding scheme presented with symptoms compatible with COVID-19 and were tested for the disease: 102 in Blantyre and 18 in Mangochi. Among these, 2 cases were confirmed in one village in Blantyre. Both cases resulted in death. There were a total of 28 deaths among those shielding in Blantyre, including those caused by COVID-19, and 30 in Mangochi. As these data were collected in an aggregated form to protect the privacy of villagers, it was not possible for researchers to disaggregate these findings further.

### Qualitative findings

***Observations***

Observations revealed that handwashing stations were available and in use at most households, although the regularly distributed soap bars were less ubiquitous. Questioning revealed that these tended to run out quickly as they were used in households for purposes other than handwashing ( *e.g*. laundry). Masks tended to be worn when traveling outside the village, to the health centre for instance, but were seen much less during day-to-day life within the village. An exception to this was in gatherings, where some adults wore masks. Gatherings were mainly outdoors, and a degree of ‘social distancing’ was also seen in some places such as schools. Handshakes (usually important in Malawian tradition) were replaced for many but not all, by fist/elbow bumps as a concession to COVID guidance. Researchers conducting observations did not find ‘COVID awareness’ materials (posters and leaflets provided by the project) on display in public areas in either of the districts, although volunteers were seen wearing branded T-shirts in both districts.

***Focus groups***

**General perceptions of the project**

When asked about the project in general there were positive responses from focus group members including residents, community leaders, and health volunteers in the villages where the project took place. Focus group participants expressed gratitude for the awareness spread by the project of COVID and prevention measures, but particularly for the resources – handwashing buckets, soap, and masks required for communities to protect themselves against COVID.

*“The people understood the project … when they came and gave the elders those pails and soap it is when it showed that this project is helpful. So, it also encouraged other people including the elders… plus looking at the other things brought up like maize flour and relish, it gave encouragement that these people are taking care of the elders.”*

Health volunteer, district level focus group, Mangochi

*“P 10: We just want to thank you, thanks a lot. In the village we face a lot of challenges so don’t stop, continue, thank you.*

*P 3: We thank you because some people just speak but you have given us like masks, soap, buckets.*

*P 8: We thank this organization that you have listened to our problems and you will pass (on) the message*

*P 6: We thank you because nowadays even at the funeral when they see a bucket, they wash their hands unlike in the past.”*

Local residents, village level focus group, Blantyre

**Perceived benefits of the project**

As indicated above, the elements of the project most positively commented upon were the addition of resources, over and above the awareness-raising and information components of the project. Focus group participants clearly explained how provision of these resources – soap, handwashing buckets, masks, and provision of food parcels for a subset of the most vulnerable through a government initiative – made it possible to enact the widely recommended COVID prevention measures.

*“Even for us who are taking care of the elderly people, it is a great thing that you are giving us soap, it is hard to have soap in the village … it could have been difficult for us to also buy soap, the price of soap nowadays has gone up”*

Resident carer for an older adult family member, village level focus group, Blantyre

*“that time they started giving the food baskets it was helpful that the person will know when I eat this, I will do this”*

Health volunteer, district level focus group, Blantyre

Suggested changes to the project

Alongside an appreciation for the various resources provided, there were frequent calls for more of these resources. Residents raised the issues of buckets breaking requiring replacement, and soap which was insufficient for all the purposes for which it was used.

This linked with a wider issue, again cited by many community members, volunteers, and staff, of a general scarcity of resources, which added complexity to the work of the project. A particular aspect of this, cited by many, was that of food security for older adults who were being encouraged to shield in their homes.

*“when you take soap and you give them, you find that the whole day she hasn’t eaten anything the whole day, and you say ‘here is the soap’”*

Health volunteer, district level focus group, Blantyre

Many focus group participants proposed donations of food and other resources to support the shielding older adults:

*“the elders were saying … apart from the fact that you are giving us soap, pails, but food is also needed in this moment since we are not travelling”*

Health volunteer, district level focus group, Mangochi

*“Considering that some elders do not have guardians and they have maybe a small (vegetable garden) or a field, right. So maybe there could be a plan to give them fertiliser and seeds, since maybe it is hard to give them maize flour... so that they might eat in the future.”*

Health volunteer, district level focus group, Mangochi

It was apparent from residents’ narratives how food insecurity prohibited some elderly individuals from shielding. In a context where insecurity was common, not all elderly residents had a named carer to consistently support their daily needs as intended in the project plans. In some cases, older people themselves had responsibility for their grandchildren, thus staying at home for protection against COVID presented additional problems. An older adult community member explained:

*“…we are staying from morning without any food. With the money problem, you would search in the house and say, in the past I would keep money in here, maybe K20, and a school child is asking you ‘granny, give me K50 so I should buy freezes at school’, and you tell them, ‘where do I get it? I don’t have (money), I am just living without doing anything.’ … there are elders just staying, some do not have children, some lost their children, they don’t have anyone to assist them.”*

Older adult community member, village level focus group, Mangochi

While the government partnership that enabled food packages to be delivered to some of the most vulnerable individuals was acknowledged, in reality many of the older adults involved in the project experienced economic scarcity and did not receive this support.

It was striking that this insecurity was not limited to older adults. Carers also brought up the need for food to support themselves in their work. One resident carer in Blantyre explained: *“You have not eaten, then you will say am tired of caring for the patient because the body is weak.”*

**Volunteer recommendations**

In focus groups, community volunteers, who were not paid for their time but received mobile phone credit to assist in their work, frequently requested more ‘benefits’ of various types, for their work on the project. Requested benefits included soap and buckets, as older adults received. One volunteer, in a village level focus group in Mangochi, stated: *“I think the volunteers should also have buckets … they are supposed to lead by example”.* Another, in Blantyre suggested, *“like us volunteers, we distribute soap and we receive airtime … but we need also soap so that we can wash”.* Other suggestions for benefits included mobile telephones to further aid communication and bicycles to fetch medicines or transport older adults to health centres, should this be necessary. Finally, there were suggestions of incentives such as refreshments during training sessions, and more high-profile venues for the sessions. As one focus group participant explained: *“in Chichewa they say if you want the cow to give you more milk, you have to give enough food so that it can give good milk”.*

The second category of recommendations from those involved in the running of the project was an extension to the scope of the project, in terms of participants, staff, and villages involved. Inclusion criteria were questioned by a few, who clearly valued the benefits afforded by the project so highly that issues of equity around which populations were involved were seen as very important. A member of a district level focus group explained: *“There are 5 pilot villages. There are so many villages complaining that they also have grandparents who are just staying and struggling, they are also old they also need that same assistance.”* Greater clarity around the selection process was a further recommendation from a few participants. This also applied to the selection of the subset of participants deemed particularly vulnerable, and to whom government food parcels was distributed.

*“the distribution of that food: the list was shortlisted and some were left out but they were supposed to give to everyone or five kgs to each and everyone could be happy. The list was shortlisted and still other people are still complaining, so this could sorted: it’s better that they should be given 5kgs but everyone should be given, that’s all.”*

Health volunteer, district level focus group, Blantyre

Further suggestions regarded the inclusion of those with certain chronic illnesses, and of additional volunteers, to cover wide geographical areas more easily.

## Discussion

In this study, we aimed to implement a COVID-19 prevention and awareness initiative, engaging communities throughout, to optimise protection for those most vulnerable to the disease. The initiative included community introduction activities aimed at community health workers and village residents, the distribution of basic WASH items, namely bucket-handwashing stations and regular soap supply, and the promotion of a voluntary shielding strategy to minimise exposure to the virus amongst those at greatest risk.

The initiative was implemented in two districts in Malawi: Blantyre, a peri-urban setting, and Mangochi, a mainly rural area. The initiative was well accepted in both settings, with the percentage of villagers over 60 years agreeing to shield themselves ranging from 25–100% and being greater than 50% in the majority of villages. Shielding of older adults required arrangements to reach their basic needs (food, cooking fuel, care, and medications), met through a designated carer arranged by families and communities themselves. In Blantyre, care for the shielded older adult was delegated to a child family member in more than half of all cases, while in Mangochi child carers were in the minority. In both districts, minor dependents who were under the care of a shielded individual remained under their care, out of necessity. In Blantyre, the economic capital of Malawi, low-skilled adults from rural villages often travel outside the village to find work, leaving children under the care of the older adult family members. In contrast, the economy of Mangochi, a rural setting, revolves around the lakeshore, making it more feasible for adults in the area to remain in their village ^27^.

To the authors’ knowledge, this is the first example of a community-led shielding strategy arranged by and sustained within the community themselves ^28^. Our work has revealed the unintended impacts that such initiatives can have for dependent minors when they are left under the care of an individual who can become vulnerable in situations such as the COVID-19 pandemic. Future pandemic preparedness plans would benefit from taking into consideration the impact of both the disease itself on the directly affected populations, and the vulnerability of those who might be left to bear the unintended consequences. Had the COVID-19 pandemic hit Malawi with the severity anticipated by early models, minors under the care of the older adult could have suffered from the loss of a main carer, while separated from parents who might not have been able to return if movement restrictions were in place ^29, 30^. These complexities demonstrate the need for ongoing engagement with communities throughout all stages of pandemic planning and response.

Qualitative evaluation found the project to be well-received amongst community members, with a range of positive feedback. The project successfully foregrounded community participation and local partnerships in its approach. A key challenge was that of structural limitations inherent to the setting – largely related to financial scarcity – which at times made it difficult for participants and volunteers to fully engage with plans.

Economic challenges in these settings were foremost in the minds of participants. While individuals were grateful for the intervention and keen to be involved, food and fuel insecurity and related issues restricted their capacity to effectively shield. This scarcity was also reflected in the response to resources supplied: even soap provided for handwashing tended to run out quickly as it was used for a variety of household purposes, and participants commonly requested that the project extend to supply food or financial support.

Volunteers’ perspectives reflected these issues. Economic incentives (and the provision of material items) were often cited as recommendations for improving the work of volunteers engaged with the project, highlighting that volunteers were operating within the same settings and subject to the same environmental stressors as other community members.

The challenges brought by COVID in Malawi, on a background of extreme scarcity and already-stretched health systems, related in part to managing the COVID disease burden whilst also mitigating its indirect economic and social effects ^11^. There is ample evidence of the COVID-related impacts on livelihoods and commodity prices, and subsequent food insecurity ^31, 32^. Results of a national survey found that 82% of participants feared going hungry following COVID-related loss of income and price impacts ^33^. Malawi also experienced worsening of various health and social outcomes due to COVID including mental health conditions, suicide, teenage pregnancy, and gender-based violence ^34^. Participants of a qualitative study in Somalia felt the indirect economic and social outcomes to be the main impact of the pandemic ^35^: a perspective echoed in participants’ responses in Malawi.

To mitigate the worst of the indirect effects of COVID, the Malawi government put in place an expanded programme of cash transfers and related financial safety nets, and a stringent national ‘lockdown’ was resisted ^11, 34^. Whilst the financial measures did have a protective effect, a recent evaluation asserted that a scaling up of such measures was required to further protect at-risk populations in Malawi ^32^.

Coetzee & Kagee use the theoretical domains framework to describe how ‘environmental context’ constrains individuals’ abilities to adhere to COVID prevention measures such as social isolation in low- and middle-income countries ^36^. This also explains how immediate concerns such as finding food and income are prioritised above seemingly less important threats such as that of COVID. The medical anthropology literature even before COVID sheds further light on tensions between underlying structural limitations and individual medical concerns. In a landmark paper, Kalofonos powerfully exposes the challenges arising during the external provision of medical solutions (antiretroviral medications for HIV) in an environment characterised by food insecurity in Mozambique, highlighting the social risks of overlooking deeper inequities in health and wellbeing whilst exclusively tending to specific medical concerns. In terms of COVID specifically, Stoler and colleagues have revealed how water insecurity in 23 countries compromised COVID responses, accentuating existing inequalities.

The Kuteteza project – incorporating a team of Malawian and international colleagues and working in partnership with national and district level structures in Malawi – made efforts to take these wider contextual factors into account throughout its planning and implementation. Despite limited resources, provision of basic resources alongside health messaging was prioritised, and cooperation with the Malawi government allowed for food packages and additional resources to be targeted to optimise equitable distribution. In this context however, the available resources proved insufficient to cover the extent of the need within communities.

Singer and Rylko-Bauer, describing the direct and indirect impacts of COVID in terms of structural violence, highlight the need for global health approaches with an “orientation toward prevention and preparation”, which address wider health determinants ^37^. Key to this is a move to real solidarity in the field of global health: attention to underlying inequities as well as ad hoc responses to emerging health issues. This is the global health challenge of our times.

### Strengths and limitations

The community-rooted nature of the project, with simple, clear messaging, and implementable recommendations (paired with the necessary resources) were a key strength of the Kuteteza project. These factors were positively reported on by participants and are and in keeping with calls in the literature for public engagement and achievable health advice to facilitate adherence ^11, 36^.

The work faced two main limitations in regards of data collection. From the implementation perspective, and given the voluntary nature of shielding, it was not possible to evaluate how faithfully participants adhered to the recommendation of limiting their social mixing. While participants were grateful for the provision of buckets and soap, and adherence to their use was evidenced through observation, we could not confirm whether participants were indeed shielding.

While the number of individuals presenting with symptoms compatible with a SARS-COV2 diagnosis was low, and only two cases were confirmed among our target population, we cannot ascertain to what extent shielding contributed to the low case load. Limitations in our data collection procedures also mean it is possible for cases to have been missed. In the project, cases were identified through records held by HSAs, who are trained in data collection procedures and trusted amongst community members ^38^ making case recording largely accurate, but national serological surveillance has revealed a SARS-COV2 circulation much greater than that reported ^39^. Colleagues have reported that up 85% of participants recruited within a Malawian cohort with positive SARS-COV2 serology were asymptomatic ^40^. This suggests – in line with serological studies and national data – that the number of symptomatic cases among shielded individuals in our intervention was likely to have been low, with total infections under-reported in our study ^39, 41^. Higher than reported circulation of the virus would also advocate for the implementation of strategies to restrict exposure of susceptible, high-risk individuals in the event of the emergence of new SARS-COV2 variants, in conjunction with other medical and non-medical interventions.

## Conclusions

In the event of health emergencies, involving communities in initiatives that contribute to their protection can effectively raise awareness, ensure the spread of scientifically-sound information and advice, as well as ensuring that preventative measures are locally appropriate and feasible. However, the design of any community-led initiative needs to be carefully considered to ensure that protecting those vulnerable to the emerging threat does not result in unintended harms within communities. In this work we witnessed how selective lockdowns in rural communities in Malawi could have resulted in children being left with the burden of having to care for older adult relatives for example.

Community health workers are trusted healthcare workers with often high levels of influence among those they serve and were instrumental in facilitating the implementation of this work. We would recommend their involvement in future community-based health preparedness and response plans, whilst noting that they may often face the same difficulties as those in their local communities and must also have their health, economic, and wider needs met as far as possible. Likewise, enhancing the use of community-based health interventions through the integration of community volunteers can also prove an invaluable tool in the prevention and early detection of health emergencies.

Finally, this project evidences the critical role of understanding local contexts in terms of pre-existing and continuing challenges experienced by communities through their daily lives. Health and development interventions – even those in emergency settings such as infectious disease outbreaks – would be strengthened by attention to, and action on, these factors.

## Ethics and consent

The original project was a piece of responsive Public Health work. Shielding was implemented as part of the Public Health response to COVID-19, in close collaboration with the Ministry of Health and local health authorities. Participation in shielding elements was voluntary, and no information was required from those who opted into shielding, other than access to anonymised population level data. In terms of the qualitative evaluation, focus group participants were required to provide written informed consent, which was collected by trained field workers, and all data and documents were kept under the custody of the principal investigator in a locked cabinet, located in a restricted-access area. A named safeguarding lead was in place throughout the project, with various avenues of contact for participants to report any concerns, and structures for appropriate referral of any such reports. The project was sponsored and approved by the local ethics committee (College of Medicine Research Ethics Committee; COMREC) in Blantyre, Malawi (P.01/21/3246) on 7 ^th^ July 2021.

## Data Availability

The quantitative and qualitative data supporting the conclusions of this article are not publicly available due to confidentiality concerns. Limited data may be made available on individual request via email to the corresponding author, with requests for additional data evaluated by the principal investigator on a case-by-case basis. Harvard Dataverse: 'Kuteteza' project - additional documents. https://doi.org/10.7910/DVN/AKVXEC ^23^ This project contains the following extended data: - Reflexivity statement - Focus group discussion guide - Additional data collection tools - Participant information sheets and consent forms Data are available under the terms of the Creative Commons Zero "No rights reserved" data waiver (CC0 1.0 Public domain dedication).
