# Supplementary material for: “Kuteteza”: A community-engaged COVID-19 Prevention and Protection Initiative in Southern Malawi
Source: Wellcome Open Res. 2024 Oct 2;9:24. Originally published 2024 Feb 12. [Version 2] doi: 10.12688/wellcomeopenres.20789.2 (PMC11514378; doi:10.12688/wellcomeopenres.20789.2)
Supplement: Peer review history

# Reviewer report: Reviewer response for version 2

**DOI:** 10.21956/wellcomeopenres.25470.r106898

**Published:** 2024-11-25

**Authors:** May Chu

**Affiliations:** 1 Colorado School of Public Health, Colorado, USA

This report provides evidence that compliance to protective measures, here, for COVID-19 can have good community uptake by providing resources like soap and buckets for hand washing, and that basic community need is in high demand. 

 One minor consideration is that the authors need to be consistent to use COVID or COVID-19, technically it should be the latter.

Is the work clearly and accurately presented and does it cite the current literature?

Yes

If applicable, is the statistical analysis and its interpretation appropriate?

Yes

Are all the source data underlying the results available to ensure full reproducibility?

Yes

Is the study design appropriate and is the work technically sound?

Yes

Are the conclusions drawn adequately supported by the results?

Yes

Are sufficient details of methods and analysis provided to allow replication by others?

Yes

Reviewer Expertise:

COVID-19 Infection prevention and control.  Infectious disease outbreak response

I confirm that I have read this submission and believe that I have an appropriate level of expertise to confirm that it is of an acceptable scientific standard.

# Reviewer report: Reviewer response for version 2

**DOI:** 10.21956/wellcomeopenres.25470.r102662

**Published:** 2024-10-25

**Authors:** Hanna Chidwick

**Affiliations:** 1 McMaster University, Hamilton, Ontario, Canada

The revised paper effectively integrated reviewer comments resulting in a clearer description and discussion of the findings.

Is the work clearly and accurately presented and does it cite the current literature?

Partly

If applicable, is the statistical analysis and its interpretation appropriate?

I cannot comment. A qualified statistician is required.

Are all the source data underlying the results available to ensure full reproducibility?

Yes

Is the study design appropriate and is the work technically sound?

Yes

Are the conclusions drawn adequately supported by the results?

Partly

Are sufficient details of methods and analysis provided to allow replication by others?

Partly

Reviewer Expertise:

Community-engaged research, East Africa, global health, vulnerable populations (adolescents, older adults), futures

I confirm that I have read this submission and believe that I have an appropriate level of expertise to confirm that it is of an acceptable scientific standard.

# Reviewer report: Reviewer response for version 2

**DOI:** 10.21956/wellcomeopenres.25470.r102661

**Published:** 2024-10-16

**Authors:** Thiago Cerqueira-Silva

**Affiliations:** 1 Instituto Gonçalo Moniz, Fiocruz, Brazil

The authors satisfactorily justified the reason for the absence of certain data. I have no new comments.

Is the work clearly and accurately presented and does it cite the current literature?

Yes

If applicable, is the statistical analysis and its interpretation appropriate?

Not applicable

Are all the source data underlying the results available to ensure full reproducibility?

Partly

Is the study design appropriate and is the work technically sound?

Yes

Are the conclusions drawn adequately supported by the results?

Yes

Are sufficient details of methods and analysis provided to allow replication by others?

Partly

Reviewer Expertise:

Epidemiological surveillance; infectious disease epidemiology

I confirm that I have read this submission and believe that I have an appropriate level of expertise to confirm that it is of an acceptable scientific standard.

# Reviewer report: Reviewer response for version 1

**DOI:** 10.21956/wellcomeopenres.23006.r76388

**Published:** 2024-05-15

**Authors:** Hanna Chidwick

**Affiliations:** 1 McMaster University, Hamilton, Ontario, Canada

An interesting study and important topic in how to prioritize community-led approaches to health interventions. I have included specific comments for consideration below. Thanks for bringing this work forward!

 The use of the term ‘shielding’ could be clarified in the abstract since it is used as a technical term. Consider using quotations in the abstract when referring to it or defining explicitly.

 The abstract seems to describe the Kuteteza project itself rather than the evaluation of the project as presented in this paper. Revisions for alignment with the qualitative and quantitative methods as well as specific study results presented in this paper would be clarifying and beneficial.

 I would be curious to know more about the experience of older adults during COVID to further justify the focus of the study. Perhaps this could be briefly expanded upon in the Introduction.

 I am somewhat confused about the objectives or aims of this study – it could be clarified that this paper outlines the evaluation of a previously implemented community-engaged shielding approach. Why the evaluation of such an approach is important could be clarified as well and incorporated into the Discussion section.

 The mention of the FGD and other study tools on p.5 after describing the community level engagement seems out of place in the “Study setting and population” section. Consider moving to the data collection or another section that is more relevant.

 How was the Kuteteza package developed? On p.5, Phase 2 mentions it was implemented but I would be curious about detail on how it was developed. This section also refers to “Local activities incorporated:…” what does this mean? Are these the activities that the package included? Clarification in language and relevance would be helpful here.

 A small note but perhaps consider the use of the term “Enumeration” on p.5. Given the discussion of community-engaged, participatory work, using the term enumeration somewhat connotes a lack of agency amongst the individuals that participated. Perhaps consider using a different term.

 On p. 5, how was the quantitative data collected? Were participants involved or was this data all collected from secondary data sources on village residence listings? How this quantitative data was collected/how individuals were involved would be interesting to expand upon as I am curious if participatory or community-engaged approaches to this were incorporated. (Note, I see later in the “Data Analysis” section it mentions quantitative data collection – this would be helpful to move to the “Data Collection” section of the paper. Were these paper-based report forms pre-tested or developed collaboratively with community?).

 On p. 5 “Ethical considerations” consider the grammar/capitalization of the term “public health”.

 Table 1 on p.6 uses the sub-heading “Participants” to refer to two columns. This should be revised and clarified.

 Was there any incentive to participate? I am curious about how the study was explained to participants in the informed consent process – is ‘shielding’ a known term? Was there any incentive to participate outside of potential health benefits?

 On p.6, why is there mention of Taguette? Since the team used NVivo, it seems odd to mention a free/open source qualitative analysis tool. Perhaps this mention of Taguettte could be contextualized in language around accessibility or clarify why it is included.

 On p. 6-7, is there gender-disaggregated data available? If not, why was the individual quantitative data not analyzed or disaggregated based on gender?

 Interesting results presented.

 On p. 10, second paragraph of the Discussion – this paragraph seems repetitive of the results and does not clearly offer a discussion or analysis of the results. Perhaps revise to highlight a clearer central point rather that repetition of geography/about the intervention.

 I was slightly confused by the Discussion section and it read as somewhat disjointed. I wonder if it could be reorganized or streamlined to more clearly articulate the lessons learned and how they are related to policy, financial scarcity, future projects etc. The Discussion notes that this project is the first example of a community-led shielding strategy – I would be curious to know more about what worked well and what didn’t work with the community leading this strategy? It seems this section jumps from how the study was received to the challenge of financial insecurity to cooperation with Malawian government. It would be helpful as a reader to know how these points connect to the overall aim of the paper (which I understand as arguing for the importance of community engagement in research) and how the learning from the study is connected to current literature about community-led health interventions generally.

 I wonder about the sustainability of Kuteteza – what happened after the implementation? Is the project still running and the government still providing resources? What considerations for sustainability of providing such resources were considered while developing and implementing the project?

 The Conclusion section was more clear and direct. I wonder about the role of elderly adults themselves? The paper argues for including communities so that interventions are context specific and not unintentionally harmful and then calls for CHWs to be involved, what about elderly people? How can they be involved? Did the evaluation of the study provide insights on what worked well or did not work well with engaging this unique population?

Is the work clearly and accurately presented and does it cite the current literature?

Partly

If applicable, is the statistical analysis and its interpretation appropriate?

I cannot comment. A qualified statistician is required.

Are all the source data underlying the results available to ensure full reproducibility?

Yes

Is the study design appropriate and is the work technically sound?

Yes

Are the conclusions drawn adequately supported by the results?

Partly

Are sufficient details of methods and analysis provided to allow replication by others?

Partly

Reviewer Expertise:

Community-engaged research, East Africa, global health, vulnerable populations (adolescents, older adults), futures

I confirm that I have read this submission and believe that I have an appropriate level of expertise to confirm that it is of an acceptable scientific standard, however I have significant reservations, as outlined above.

# Response

**Published:** 2024-09-12

**Authors:** Sepeedeh Saleh

**Affiliations:** Malawi-Liverpool-Wellcome Trust Clinical Research Programme, Blantyre, Southern Region, Malawi

Dear Dr Chidwick,

 Many thanks for your review of this paper, which has now been revised in response to the comments.   We have reproduced and addressed individual comments below (reviewer comments in italics).

***The use of the term ‘shielding’ could be clarified in the abstract since it is used as a technical term.* *Consider using quotations in the abstract when referring to it or defining explicitly.***

**Response:** Thank you for this suggestion. This has been amended throughout the text.  

***The abstract seems to describe the Kuteteza project itself rather than the evaluation of the project as presented in this paper. Revisions for alignment with the qualitative and quantitative methods as well as specific study results presented in this paper would be clarifying and beneficial.*** 

**Response:** Thank you for this observation. The abstract has been rewritten in response to this comment  

***I would be curious to know more about the experience of older adults during COVID to further justify the focus of the study. Perhaps this could be briefly expanded upon in the Introduction*.**

**Response:** Many thanks for this interesting question. Unfortunately, as more of an immediate response to the threat of COVID in the older population, this project did not provide the wider background concerning the experiences of older adults in the pandemic in Malawi. We agree that this would be a fascinating and important area of enquiry for a qualitative research project.  

***I am somewhat confused about the objectives or aims of this study – it could be clarified that this paper outlines the evaluation of a previously implemented community-engaged shielding approach. Why the evaluation of such an approach is important could be clarified as well and incorporated into the Discussion section.***

**Response: **Many thanks Dr Chidwick for raising this concern. This has now been clarified throughout the abstract and in the background and results sections, and is mentioned in the Discussion – for instance at the start of the ‘Strengths and Limitations’ subheading.  

***The mention of the FGD and other study tools on p.5 after describing the community level engagement seems out of place in the “Study setting and population” section. Consider moving to the data collection or another section that is more relevant.***

**Response: **Thank you for highlighting this. It has now been moved as suggested.  

***How was the Kuteteza package developed? On p.5, Phase 2 mentions it was implemented but I would be curious about detail on how it was developed.***

**Response: **Many thanks for your interest in this. The Kuteteza project was envisioned on the basis of proposed guidance published early in the pandemic on the shielding concept as applied to refugee camps ( https://www.lshtm.ac.uk/media/38941). In view of the breadth of local knowledge and experience existing within our team, we adapted the approach making it contextually relevant and applicable to rural Malawian settings. A multidisciplinary team of researchers worked with in-country Public Health systems and non-governmental organisations, and with the Ministry of Health to coordinate the response and avoid duplication of efforts. We co-developed plans together with community volunteers and community health workers, also engaging the public through community leaders, to understand the immediate and evolving needs. The project integrated timely dissemination of reliable, evidence-based information regarding COVID-19 and the status of the pandemic, and two-way feedback between community members and elements of the community health system. A key consideration for the project was to ensure all information and materials were distributed in Chichewa, the local language, and that all community-facing activities were conducted by local staff, including by village residents themselves when appropriate.   We have endeavoured to include the main principles of this throughout the paper, and have tried to clarify this in the current version, but the details of intervention development unfortunately lie outside the scope of this publication (as you correctly identified – the focus of the current paper is the project evaluation).  

***This section also refers to “Local activities incorporated:…” what does this mean? Are these the activities that the package included? Clarification in language and relevance would be helpful here.***

**Response: **Many thanks for this query – the point in question has been rephrased for clarity.  

***A small note but perhaps consider the use of the term “Enumeration” on p.5. Given the discussion of community-engaged, participatory work, using the term enumeration somewhat connotes a lack of agency amongst the individuals that participated. Perhaps consider using a different term.***

**Response: **Thank you also for this point – the text has been revised in line with the comments. *On p. 5, how was the quantitative data collected?*

***Were participants involved or was this data all collected from secondary data sources on village residence listings? How this quantitative data was collected/how individuals were involved would be interesting to expand upon as I am curious if participatory or community-engaged approaches to this were incorporated. (Note, I see later in the “Data Analysis” section it mentions quantitative data collection – this would be helpful to move to the “Data Collection” section of the paper. Were these paper-based report forms pre-tested or developed collaboratively with community?)*******

**Response: **Thank you for this request for clarification. Aggregated data were requested and collected by project staff from the community health workers and recorded in dedicated data forms. While these quantitative data were gathered in partnership with local community health workers, community members themselves were not involved in their collection. As this project mainly aimed to demonstrate feasibility, such details were not a core part of the research and were not classed as part of the participatory elements of the project. Vaughn and Jacquez in their account pf participatory research describe the range of participatory approaches and the various stages of research at which they might be applied [ https://doi.org/10.35844/001c.13244].   We are grateful also for your bringing our attention to the issue in the data analysis section. This text has now been clarified such that descriptions appear in the correct sections.  

***On p. 5 “Ethical considerations” consider the grammar/capitalization of the term “public health”.***

**Response: **Thank you. This has been amended throughout the text.  

***Table 1 on p.6 uses the sub-heading “Participants” to refer to two columns. This should be revised and clarified.***

**Response: **Thank you for pointing out this error, which has now been revised.  

***Was there any incentive to participate? I am curious about how the study was explained to participants in the informed consent process – is ‘shielding’ a known term? Was there any incentive to participate outside of potential health benefits?***

**Response: **Thank you for your interest in these issues. Participation in shielding was voluntary and compliance was not monitored. The preparatory work and awareness-raising component (p.10) lay the groundwork for community understanding of COVID risks and transmission, allowing engagement with the principles of shielding. There was no monetary incentive for taking part. However, those who identified themselves as over 60 years of age and expressed their willingness to minimise their social interactions were provided with a Veronica bucket that they placed at the entrance of their home and a cloth face mask and were regularly supplied with soap.  

***On p.6, why is there mention of Taguette? Since the team used NVivo, it seems odd to mention a free/open source qualitative analysis tool. Perhaps this mention of Taguettte could be contextualized in language around accessibility or clarify why it is included.*** 

**Response: **Thank you for highlighting this issue. Taguette was mentioned in response to Wellcome Open requirements around provision of open access alternatives to any software mentioned. The wording around this has been amended but we are open to better ways of incorporating this in the text and will raise with the WO editors also.  

***On p. 6-7, is there gender-disaggregated data available? If not, why was the individual quantitative data not analyzed or disaggregated based on gender?***

**Response: **Thank you for this suggestion. The additional information has been added to table 2 and noted in the results. The majority of participants were female in both districts (61.3% overall), although gender participation was slightly more balanced in Mangochi than in Blantyre.  

***On p. 10, second paragraph of the Discussion – this paragraph seems repetitive of the results and does not clearly offer a discussion or analysis of the results. Perhaps revise to highlight a clearer central point rather that repetition of geography/about the intervention.***

**Response: **Many thanks. This paragraph has now been rewritten to resolve the issue.  

***I was slightly confused by the Discussion section and it read as somewhat disjointed. I wonder if it could be reorganized or streamlined to more clearly articulate the lessons learned and how they are related to policy, financial scarcity, future projects etc. The Discussion notes that this project is the first example of a community-led shielding strategy – I would be curious to know more about what worked well and what didn’t work with the community leading this strategy? It seems this section jumps from how the study was received to the challenge of financial insecurity to cooperation with Malawian government. It would be helpful as a reader to know how these points connect to the overall aim of the paper (which I understand as arguing for the importance of community engagement in research) and how the learning from the study is connected to current literature about community-led health interventions generally.***

**Response: **Thank you for this analysis. The discussion section has been rewritten in line with the advice, to add clarity and to improve the communication of the main messages of the paper. We agree that this project serves as an example of the importance of community engagement in research, but issues of local relevance and engagement with local context – including government, health and public health infrastructure in this case – are also important here, hence the involvement of these literatures in the discussion.  

***I wonder about the sustainability of Kuteteza – what happened after the implementation? Is the project still running and the government still providing resources? What considerations for sustainability of providing such resources were considered while developing and implementing the project?***

**Response:** We are grateful for the reviewers’ interest in this element of the project. The shielding initiative conceptualised as the “Kuteteza” project was incorporated into the broader national response to COVID in a pilot that took place across several villages in two Malawian districts. It was part of a Ministry of Health-led response package and was not intended as an initiative to be sustained over time, but as an approach to minimising the risks of COVID for older adults during high transmission periods. The provision of food packages and economic support were the responsibility of the Malawian government, and the project did not intervene in these elements. The project did, however, supply soap and masks, and supported the dissemination of information materials, which the Ministry of Health was free to distribute nationally and beyond pilot villages, again during the acute phase of the pandemic. These elements were all sustained during the key periods of the pandemic but were not intended to continue beyond these periods and thus sustainability in this sense was not planned for.

***I wonder about the role of elderly adults themselves? The paper argues for including communities so that interventions are context specific and not unintentionally harmful and then calls for CHWs to be involved, what about elderly people? How can they be involved? Did the evaluation of the study provide insights on what worked well or did not work well with engaging this unique population?*******

**Response: **We are grateful to the reviewers for this interesting question. Community volunteers, community health workers, and village leaders and residents (young and old) were actively engaged in the project from its conception and their input was integrated in intervention development and implementation. Village residents were key to ensuring that all activities were culturally appropriate, and that health messages could be well understood and received, as well as feeding back community perspectives around COVID and the project, which were built on as implementation progressed. The paper focuses mainly on the feasibility, successes and challenges of this community-based integrated ‘shielding’ initiative in Malawi, unfortunately precluding more specific discussion of the successes and challenges of engaging with older residents in particular. We agree that this would make a useful topic for future exploration.  Once again, we thank you for your engagement with the paper and for the thorough and insightful review, which we believe has led to a much-improved manuscript.    

 Kind regards

 Dr Sepeedeh Saleh MBChB, MPH, MFPH, DTMH, PhD, on behalf of the ‘Kuteteza’ team

# Reviewer report: Reviewer response for version 1

**DOI:** 10.21956/wellcomeopenres.23006.r76392

**Published:** 2024-04-10

**Authors:** Thiago Cerqueira-Silva

**Affiliations:** 1 Instituto Gonçalo Moniz, Fiocruz, Brazil

It is an interesting project on how to manage COVID-19 during the pandemic in a resource-constrained country. My main comments are only about the methods; if possible, examples of the materials used to train volunteers, such as posters and leaflets, could be offered as supplementary material, making it easier for other communities to replicate the work.

 In the results, the description of the number of individuals should include a rough estimate of the total population of the 25 villages, allowing us to assess the overall impact. An additional image with a cartogram depicting the villages represented in the study is also beneficial to those unfamiliar with Malawi. In table 2, the phrase "individuals with data" is not particularly apparent. Table 3 appears to be disordered (the division between Blantyre and Mangochi is not clearly labelled).

 Is there a comparison between the characteristics of those who accepted to engage in the shielding and those who did not?

 Is it possible to compare the total number of deaths and only deaths due to COVID-19 in the villages under the Kuteteza and those without the programme? It would allow estimate an indirect effect of the programme.

Is the work clearly and accurately presented and does it cite the current literature?

Yes

If applicable, is the statistical analysis and its interpretation appropriate?

Not applicable

Are all the source data underlying the results available to ensure full reproducibility?

Partly

Is the study design appropriate and is the work technically sound?

Yes

Are the conclusions drawn adequately supported by the results?

Yes

Are sufficient details of methods and analysis provided to allow replication by others?

Partly

Reviewer Expertise:

Epidemiological surveillance; infectious disease epidemiology

I confirm that I have read this submission and believe that I have an appropriate level of expertise to confirm that it is of an acceptable scientific standard, however I have significant reservations, as outlined above.

# Response

**Published:** 2024-09-12

**Authors:** Sepeedeh Saleh

**Affiliations:** Malawi-Liverpool-Wellcome Trust Clinical Research Programme, Blantyre, Southern Region, Malawi

Dear Dr Cerqueira-Silva,

 Many thanks for your review of this paper, which has now been revised in response to the comments.   We have reproduced and addressed individual comments below (reviewer comments in italics).

***If possible, examples of the materials used to train volunteers, such as posters and leaflets, could be offered as supplementary material, making it easier for other communities to replicate the work***

**Response: **Thank you for this suggestion. Posters and leaflets have now been added as supplementary materials.  

***In the results, the description of the number of individuals should include a rough estimate of the total population of the 25 villages, allowing us to assess the overall impact. An additional image with a cartogram depicting the villages represented in the study is also beneficial to those unfamiliar with Malawi***

**Response: **Thank you. We appreciate the wish to understand the extent of impact in terms of proportions of village residents participating. Unfortunately, due to the poor quality of demographic information systems in Malawi and the dynamic and transient nature of certain rural populations – with many younger residents working in urban areas for extended periods, for example – it is impossible to provide the requested data with any degree of confidence. Similarly, mapping of rural villages in Malawi is not available: such maps only extend to district level, with maps showing the two districts in question available from a range of sources online.

***In table 2, the phrase "individuals with data" is not particularly apparent.***  

**Response: **Many thanks for pointing this out. This wording has been altered in the table for clarity, and gender has also been added to increase the comprehensiveness of the data provided.  

***Table 3 appears to be disordered (the division between Blantyre and Mangochi is not clearly labelled)*******

**Response: **Thank you also for raising this issue, which has now been addressed in the table  

***Is there a comparison between the characteristics of those who accepted to engage in the shielding and those who did not?***

**Response: **Thank you, Dr Cerqueira-Silva, for this question. While researchers agree that this would have been useful information to have, due to the need for quick response, we did not request any information from eligible villagers at the time Kuteteza was set up. Participation was completely voluntary, and the project simply requested those aged 60 years or over to limit their social contacts on a voluntary basis. We did not, in fact, control adherence nor collect any individual information from villagers, regardless of their participation status, as this would have required a detailed ethical clearance process and the application of informed consent which, at the time of a global pandemic and great uncertainty in Malawi, we considered not to be practical.  

***Is it possible to compare the total number of deaths and only deaths due to COVID-19 in the villages under the Kuteteza and those without the programme? It would allow estimate an indirect effect of the programme.***

**Response: **We thank the reviewer for this pertinent question. Shielding was implemented as one of several components of a package, and as a complement to national Public Health response measures directed by the Malawi Ministry of Health. All collected data collected were anonymised, and only aggregated health data were obtained from Health Surveillance Assistants. Hence, it was not possible to disaggregate data between shielded and unshielded individuals. As experience in the Northern hemisphere had indicated that preventing social contact, and therefore virus transmission, could present an effective way of protecting vulnerable individuals, the project was mainly conceived to evaluate the feasibility of the approach. Neither the intervention nor the current evaluation were designed, or statistically powered, to measure quantitative impacts on COVID-19 case numbers or outcomes.

 However, we agree that, had time and circumstances allowed, this would have been important information to collect. This will be a key consideration should this approach be implemented on a larger scale in the future.   Once again, we thank you for your appreciation of the paper content and for the thorough and insightful review.    

 Kind regards     Dr Sepeedeh Saleh MBChB, MPH, MFPH, DTMH, PhD, on behalf of the ‘Kuteteza’ team
